# ﻿Revision of the macropterous subgenus *Curtonotus* from east China, with the description of a new species (Carabidae, Zabrini, Amara)

**DOI:** 10.3897/zookeys.1190.109539

**Published:** 2024-01-22

**Authors:** Yihang Li, Haoyuan Li, Hongliang Shi

**Affiliations:** 1 College of Agriculture, Purdue University, West Lafayette, Indiana 47906, USA Purdue University West Lafayette United States of America; 2 College of Life Science, Capital Normal University, Beijing 100048, China Capital Normal University Beijing China; 3 College of Forestry, Beijing Forestry University, Beijing 100083, China Beijing Forestry University Beijing China

**Keywords:** Coleoptera, key, secondary sexual characteristics

## Abstract

Species from east China belonging to the subgenus Curtonotus were studied, resulting in the description of a new species, Amara (Curtonotus) beijingensis**sp. nov.** The type locality is Xiaolongmen Forest Park in Beijing. All the known macropterous *Curtonotus* species from eastern China are reviewed and for each species taxonomical notes, illustrations, and new provincial records are noted. An improved key for their identification is provided as well.

## ﻿Introduction

Ground beetles belonging to AmaraBonelli, 1810subgenusCurtonotus Stephens, 1827, are commonly observed in various provinces of China. They are easily distinguished from representatives of the other subgenera of the genus by their relatively large size and constricted pronotum base. The subgenus has a Holarctic distribution and is especially diverse in China, with a total of 94 valid species known so far (Anichtchenko 2023). Among these, 15 species are found in the Nearctic Realm (seven of them also present in the Palearctic Realm), while 79 species are confined to the Palearctic Realm (Hieke 2017). In total, 41 *Curtonotus* species are recorded from China, with many of them being widely distributed in the northern regions and others restricted to the southwestern mountainous areas.

A recent taxonomic revision of Chinese subgenus Curtonotus published by Hieke (2010) defined two main groups and described eight new species. Since then, no further new *Curtonotus* species have been reported from China. Recently, during the examination of a large number of carabid specimens collected from the Xiaolongmen Forest Park in Beijing, an undescribed species of *Curtonotus* was found. This species is most similar to A. (C.) macronota Solsky, 1875, but differs significantly in the shape of the apical lamella of male genitalia. While examining this material, several interesting records of *Curtonotus* from China were also discovered.

In this paper, we present the description of a new species of the subgenus Curtonotus found in Beijing. Additionally, we provide detailed notes on all the “macropterous *Curtonotus*” species documented in eastern China. The new species is thoroughly characterized through extensive descriptions and illustrations. For other macropterous *Curtonotus* species recorded in eastern China, we offer identification keys and illustrations, except in cases where materials were unavailable. The primary purpose of the current work is the identification of all macropterous *Curtonotus* species known in eastern China, encompassing all provinces except Xinjiang and Xizang.

It is worth noting that the males of most species from subgenus Curtonotus exhibit modified mesotibiae. In light of this, we represent a brief discussion for male mesotibiae projections. This sexually dimorphic trait not only holds taxonomical significance but also potentially plays a role in copulation and might be linked to sexual conflicts between the sexes.

## ﻿Material and methods

This work was based primarily on the examination of specimens from China. Institutional and private collections cited in the present paper are indicated by the following abbreviations:

**IZAS**Institute of Zoology, Chinese Academy of Sciences, Beijing, China;

**CBJFU**Forest Entomology Laboratory, Beijing Forestry University, Beijing, China;

**CLHY** collection of Haoyuan Li, Beijing, China;

**CLYH** collection of Yihang Li, Beijing, China;

**CCJH** collection of Jiaheng Chen, Puning, Guangdong, China;

**CUMZ**University Museum of Zoology, Cambridge, United Kingdom;

**CMNC**Canadian Museum of Nature, Ottawa, Canada;

**ITLJ**National Institute of Agro-environmental Sciences, Tsukuba, Ibaraki, Japan;

**MNHN**Muséum National d’Histoire Naturelle, Paris, France;

**MNHU**Museum für Naturkunde der Humboldt-Universität zu Berlin, Berlin, Germany;

**MSNG**Museo Civico di Storia Naturale (Giacomo Doria), Genova, Italy;

**NHML**Natural History Museum, London, United Kingdom;

**NHRS**Naturhistoriska Rihsmuseet, Stockholm, Sweden;

**NMPC**Narodni Muzeum Prirodovedecke Muzeum, Prague, Czech Republic;

**TMB** Természettudományi Múzeum Allattara, Budapest, Hungary;

**ZRAS** Zoological Institute, Russian Academy of Science, St. Petersburg, Russia;

**ZMUC**Zoological Museum, University of Copenhagen, Copenhagen, Denmark;

**ZMUM**Moscow State University, Moscow, Russia.

Male genitalia were dissected using fine forceps and glued on mounting cards. For each species, illustrations of left-lateral and dorsal views of the median lobe of aedeagus and dorsal view of right paramere are provided for one specimen of each species. The gonocoxites of ovipositors were pulled out using fine forceps but not removed from the apex of the abdomen.

Most morphological terms in the present paper follow their general applications. When referring to the orientation of the median lobe of male genitalia, “left” or “right” was determined in dorsal view with the apex of the median lobe pointing anteriorly and the base ventrally.

Measurements and abbreviations are as follows: ,length of body (**BL**), the linear distance from the apex of labrum to elytral sutural apex; ,head maximum width (**HW**) across the outer margin of eyes; ,pronotum maximum width (**PW**); ,pronotum length (**PL**), measured along median line; ,pronotum anterior width measured along tips of anterior angles (**PAW**); ,pronotum basal width measured along tips of posterior angles (**PBW**); ,elytra length (**EL**), the linear distance from apex of scutellum to elytra sutural apex; ,elytra width (**EW**) maximum width of elytra.

For each taxon, original and important taxonomic references are cited. Genus combination, information on the name-bearing types, newly recorded localities, and other important comments are listed in parentheses after each reference. Newly recorded localities are labeled with an asterisk. Full-body photographs of all species were captured by a Nikon D7200 camera with LAOWA 60 mm F2.8 2:1 Super Macro lens; male genitalia, pronotum, mesotibial projections, and female ovipositors were captured by the same camera with a LAOWA 25 mm F2.8 2.5–5X Ultra Macro lens. For each final image, several photographs were taken at different focal planes, combined with ZereneStacker software to obtain one synthesized photograph, and finally edited by Photoshop Elements 2022 Editor 20.0.

## ﻿Taxonomic account

### ﻿Subgenus Curtonotus

Taxon classificationAnimaliaColeopteraCarabidae

Stephens, 1827

289D2FC6-3CA8-53F5-BFF1-04AC5367BDAC

#### Type species.

*Amaraconvexiuscula* (Marsham, 1802); type locality: “England”.

The subgenus Curtonotus is recognizable among genus *Amara* by the combination of the following characteristics: 1) medium to large sized species (7–25 mm); 2) prosternal process not bordered, without setae at apex; 3) inner margin of male mesotibiae with projection in most species (present as one to three distinct denticles); 4) right paramere without a terminal hook; 5) pronotum more or less cordate, constricted to base, distinctly narrower than elytral base; 6) mesofemora mostly with only two posterior setae.

The subgenus Curtonotus was regarded as the most basal clade of the tribe Zabrini (Sánchez-Gea et al. 2004), but the intra-group relationships among the species of *Curtonotus* are still unclear. *Curtonotus* is similar to the subgenera *Bradytulus* Tschitscherine and *Tibetamara* Makarov & Sundukov in external appearances (Hieke 2010; Makarov and Sundukov 2021). The subgenus Bradytulus is different from *Curtonotus* by the generally much smaller body size, prosternal process with reduced border, and male metatibiae mostly with tufted ensiform setae. *Tibetamara* is different from *Curtonotus* by the male mesotibiae without a projection, right paramere shortened, and a relatively wide pronotum base. Some large species of the subgenus Bradytus may also be similar to *Curtonotus* but can be distinguished by prosternal process bordered, grooves of pronotum basal fovea shorter and usually shallower, and the right paramere usually with an apical hook. The subgenera *Amathitis* Zimmermann, *Ammoleirus* Tschitscherine, *Cribramara* Kryzhanovskij, *Harpalodema* Reitter, and *Hyalamara* Tschitscherine also have a constricted pronotum base, but can be readily distinguished from *Curtonotus* by having multiple setae on the mesofemora.

*Curtonotus* species can be found in various open habitats, including grassland, alpine meadow, coastal areas, riparian flood plains, riverbanks, and forest margins. Throughout our observations, we noted that many inhabit similar environments together with other *Amara* and *Harpalus* Latreille species. Both adults and larvae of *Curtonotus* demonstrate omnivorous feeding habits, consuming other insect larvae as well as plant seeds (Sasakawa 2010). Among them, species like A. (C.) gigantea Motschulsky, 1844 are aggressive predators. Macropterous species can be attracted by light sources, and some species climb on stalks or leaves for foraging. Certain widely distributed species, like A. (C.) gigantea and A. (C.) macronota, are commonly found in urban areas and agrarian lands, suggesting potential roles in pest and weed control (Sasakawa 2009). Moreover, other species such as A. (C.) brevicollis Chaudoir, 1850, A. (C.) dux Tschitscherine, 1894, and A. (C.) fodinae Mannerheim, 1825 have also been recorded in proximity to residential areas.

According to Hieke’s (2010) work, the Chinese *Curtonotus* species were classified into two groups, mainly based on the shape of metepisternum. Group A includes the macropterous *Curtonotus*, with hind wings usually well developed (rarely shortened), metepisternum long, with length on outer margin nearly twice as long as the anterior width. Most species of this group have widespread distributional ranges, more commonly seen in the northern and eastern provinces of China. The Group B contains brachypterous *Curtonotus*, with hind wings usually rudimentary (rarely shortened); metepisternum short, with length on outer margin subequal to the anterior width. Most species of this group are narrowly distributed, only known from the high mountain areas of west China.

In the present paper, we focus on the macropterous *Curtonotus* from the eastern provinces of China with describing a new species and providing supplementary notes on all other species so far recorded in this area. Based on available materials, four species groups of the macropterous *Curtonotus* were recognized in the eastern China: *gigantea* group, *tumida* group, *brevicollis* group, and *macronota* group, mainly based on the characteristics of head size, number of supraorbital setae, shape of pronotum, male mesotibiae projection, and the apex of male genitalia. Other *Curtonotus* species not recorded from this area may have relationships with some of the above species groups, but they have not been treated in the present paper.

### ﻿Key to the macropterous species of the subgenus Curtonotus from central and eastern China

**Table:** 

1	Larger species, body length > 16 mm; male mesotibiae projection composed of a very large triangular proximal denticle and two small distal denticles	**A. (C.) gigantea (*gigantea* group)**
–	Smaller species, body length < 14 mm; male mesotibiae projection not as above, if with triangular proximal denticle, only one distal denticle present	**2**
2	Dorsum reddish brown, elytra usually with faint bronze luster; pronotum with fine and dense punctures except at middle	**3 (*brevicollis* group)**
–	Dorsum black or dark brown; pronotum mid-anterior area without or with large and sparse punctures, mostly concentrated at middle; pronotum lateral sides impunctate or with punctures confined in lateral grooves	**4**
3	Pronotum mid-lateral setae present; posterior angles acute and distinctly laterally protruded (Fig. 5B); male mesotibiae gradually swollen near middle, without definite proximal denticle; apical lamella of aedeagus longer, rounded-truncated at apex (Fig. 5F)	**A. (C.) dux**
–	Pronotum mid-lateral setae absent; posterior angles usually obtuse or nearly rectangular, much less protruded than previous species (Fig. 6B); male mesotibiae with distinct proximal denticle near middle; apical lamella of aedeagus shorter, rounded-triangular at apex (Fig. 6F)	**A. (C.) brevicollis**
4	Head with one supraorbital seta (posterior one absent); pronotum mid-lateral setae always absent	**5 (*tumida* group)**
–	Head with two supraorbital setae; pronotum mid-lateral setae present	**9 (*macronota* group)**
5	Pronotum lateral margins almost straight near posterior angles; lateral sides of abdomen sternites without punctures or wrinkles	**A. (C.) hyperborea**
–	Pronotum lateral margins distinctly sinuate near posterior angles; lateral sides of abdomen sternites punctate or wrinkled	**6**
6	Pronotum lateral grooves a little deeper, distinctly punctate (Fig. 3B)	**A. (C.) gansuensis**
–	Pronotum lateral grooves shallower, at most very sparsely punctate	**7**
7	Pronotum lateral margins moderately sinuate before posterior angles (Fig. 4B); apical lamella of aedeagus nearly straight (Fig. 4F); female gonocoxite 2 (Fig. 4G) shorter and wider, approx. 1.6 times as long as wide	**A. (C.) goniodera**
–	Pronotum lateral margin strongly sinuate before posterior angles (Habu 1953: fig. 3); apical lamella of aedeagus more or less deflected to right; female gonocoxite 2 longer, 2.0–2.3× as long as wide	**8**
8	Pronotum widest after middle; apical lamella of aedeagus longer	**A. (C.) tumida**
–	Pronotum widest near middle; apical lamella shorter	**A. (C.) shinanensis**
9	Elytra without or only with very faint microsculpture after middle; male mesotibiae with three denticles (Fig. 9C)	**A. (C.) hiogoensis**
–	Elytra with distinct isodiametric microsculpture; male mesotibiae at most with two denticles	**10**
10	Pronotum strongly constricted to base, widest point near anterior third (Fig. 8B); elytra with very strong isodiametric microsculpture; male mesotibiae projection composed of two distinct denticles, the proximal one very large and near middle of tibia (Fig. 8C)	**A. (C.) banghaasi**
–	Pronotum less constricted to base, widest point near middle; elytra with distinct isodiametric microsculpture, but shallower than in previous species; male mesotibiae projection in a different form, if composed of two denticles, the proximal one near apical third of tibia	**11**
11	Pronotum lateral margins faintly sinuate before posterior angles; posterior angles slightly laterally protruded (Fig. 1B); apical lamella of aedeagus rounded, slightly declined to right side (Fig. 1F)	**A. (C.) beijingensis sp. nov.**
–	Pronotum lateral margins distinctly sinuate before posterior angles; posterior angles strongly laterally protruded; apical lamella of aedeagus rounded-triangular, not declined to right side (Fig. 11F)	**12**
12	Pronotum mid-anterior region impunctate (Fig. 7B); male mesotibiae slightly bent at base, gradually swollen near middle, without well-defined denticles (Fig. 7C); apex of gonocoxite 2 attenuate (Fig. 7G)	**A. (C.) fodinae**
–	Pronotum mid-anterior region sparsely punctate; male mesotibiae with two well-defined denticles (Figs 10C, 11C); apex of gonocoxite 2 widely rounded (Figs 10G, 11G)	**13**
13	Pronotum more distinctly narrowed to base; outer ridge of pronotal basal fovea strongly convex; pronotum basal region and elytra striae with very coarse punctures (Fig. 11B); gonocoxite 2 of ovipositor with length subequal to greatest width (Fig. 11G)	**A. (C.) macronota**
–	Pronotum less narrowed to base; outer ridge of pronotal basal fovea moderately convex; pronotum basal region and elytra striae with finer punctures (Fig. 10B); gonocoxite 2 of ovipositor much longer than greatest width (Fig. 10G)	**A. (C.) harpaloides**

### ﻿New species descriptions

### ﻿ Amara (Curtonotus) beijingensis sp. nov.

Taxon classificationAnimaliaColeopteraCarabidae

E034804E-B2CF-548A-A103-A18D41297D2F

https://zoobank.org/AAEEA378-8EC4-4967-B5F3-1A71916DBCB9

Fig. 1

#### Type material.

***Holotype***: male (IZAS), Beijing, Mentougou District, Xiaolongmen Forest Park, 1100 m, 2018.08.16–21 [in Chinese]. ***Paratypes***, a total of 13 males and 29 females (6 males, 15 females in IZAS, 7 males, 14 females in CBJFU), all with the same localities as holotype, but in different collecting date: 2 females, 2014.08.17–22; 5 males, 7 females, 2015.08.16–21; 2 males, 11 females, 2016.08.17–22; 1 male, 1 female, 2017.08.16–21; 1 male, 1 female, 2018.08.17–22; 1 female, 2019.08.16–21; 5 females, 2021.08.16–21; 4 males, 1 female, 2022.08.17–23.

**Figure 1.**
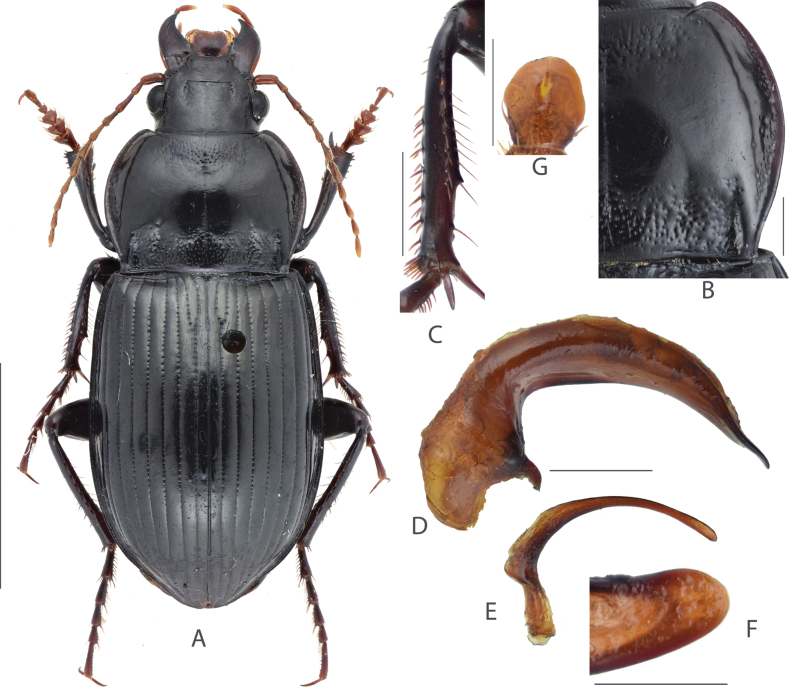
Amara (Curtonotus) beijingensis sp. nov. **A** dorsal habitus, male (holotype) **B** pronotum posterior angle **C** male mesotibia **D** lateral view of aedeagus **E** right paramere **F** dorsal view of apical lamella of aedeagus **G** gonocoxite 2 of ovipositor. Scale bars: 5 mm (**A**); 1 mm (**B–E**); 0.5 mm (**F, G**).

#### Chinese common name.

北京暗步甲.

#### Diagnosis.

A species from the *macronota* species group, with black dorsum, relatively wide head, and with two supraorbital setae; pronotum widest a little before middle; lateral margins evenly curved, narrowed and slightly sinuate before posterior angles; posterior angles evidently protruded laterally; mid-anterior area densely punctate; elytra striae distinctly punctate except near to apex; male mesotibial projection distinct, composed of two denticles: the proximal denticle is larger, its basal margin extended to form a wide triangular projection; the distal denticle is much smaller (~ 1/3 length of the proximal one), near midpoint between the proximal denticle and tibial apex; apical lamella of male genitalia large, slightly declined to right, apex rounded.

#### Comparison.

Among the Chinese species of subgenus Curtonotus, A. (C.) beijingensis sp. nov. is most similar to A. (C.) macronota for the following characteristics: two supraorbital setae present, pronotum basal area and elytral striae both distinctly punctate; pronotum anterior angles slightly protruded; male mesotibiae with two distinct denticles on inner margin; gonocoxite 2 of ovipositor very short. However, the new species is different from A. (C.) macronota in the following characteristics: elytral striae, pronotum basal and mid-anterior area with fine and dense punctures; pronotum lateral margins very slightly sinuate before the posterior angles which are less laterally protruded; elytra basal border curved upward, and apical lamella of median lobe much longer and wider, and more evidently declined to the right side, in dorsal view, apex widely rounded but not attenuate. In contrast, A. (C.) macronota has much coarser and denser punctures on elytral striae, pronotum basal and mid-anterior area; pronotum lateral margins evidently sinuate before the posterior angles which are clearly protruded laterally; elytra basal border nearly straight; and male genitalia with the apical lamella rounded-triangular, evidently attenuate to apex, not declined to the right side.

The new species A. (C.) beijingensis resembles other two species from China, A. (C.) hiogoensis Bates, 1873 and A. (C.) harpaloides Dejean, 1828. Compared with the new species, A. (C.) hiogoensis can be distinguished in the elytra with very indistinct microsculpture; pronotum without punctures on the mid-anterior area; and gonocoxite 2 of ovipositor a little longer. A. (C.) harpaloides can be distinguished from the new species in the finer punctures on pronotum and elytral striae; apical lamella of male genitalia different in shape; gonocoxite 2 of ovipositor much longer; and female with stronger elytra microsculpture.

There are six other species (A. (C.) brevicollis, A. (C.) dux, A. (C.) gansuensis Jedlicka, 1957, A. (C.) banghaasi Baliani, 1933, A. (C.) fodinae, and A. (C.) harpaloides) of *Curtonotus* in the mountain region situated west of Beijing, which may live in sympatry with A. (C.) beijingensis sp. nov. Compared with the new species, *A. (C.) brevicollis* and A. (C.) dux are different in the reddish brown dorsal surface and very fine pronotal punctures; A. (C.) gansuensis is different in the head with only one supraorbital seta; A. (C.) banghaasi is different in the pronotum widest point far before middle and anterior angles not protruded; A. (C.) fodinae is different in narrower pronotum and pronotum lateral margin curved longer before posterior angle.

We also compared the new species with all *Curtonotus* species recorded from nearby countries including Russia, Japan, Mongolia, North Korea and South Korea. A. (C.) beijingensis sp. nov. can be distinguished from most of these species by the combination of following characteristics: head with two supraorbital setae; antennomere 1 in similar color as rest segments; pronotum widest near middle, posterior angles acute and laterally protruded; pronotum disc with distinct punctures confined in the mid-anterior area. Among these species, A. (C.) beijingensis sp. nov. is externally very similar to A. (C.) gebleri Dejean, 1831 Dejean which was recorded from Mongolia and the Russian Far East. A. (C.) gebleri is different from the new species in the head a little more thickened; apical lamella of male genitalia rounded-triangular, gradually attenuate to apex, with length lesser than greatest width; gonocoxite 2 of ovipositor much more elongate. A. (C.) beijingensis sp. nov. is also similar to A. (C.) propinqua Ménétriés, 1832 which was recorded from Mongolia and Middle Asia. A. (C.) propinqua is different from the new species in the pronotum widest point far before middle; pronotum mid-anterior area only with very scarce punctures; right margin of the median lobe of male genitalia strongly swollen at middle.

#### Description.

(Habitus in Fig. 1A) Size relatively large in the genus, BL = 13.0–14.6 mm, body form rather robust; dorsum black, elytra black with shinny surface; antennae, mouthparts and tarsomeres dark reddish brown; venter black. ***Head*** relatively thick, distinctly shorter than pronotum (HW/PW = 0.5–0.6); frons sparsely wrinkled, frontal fovea short, reaching middle level of eyes; with two supraorbital setae; eyes large, hemispheric; antennae nearly reaching pronotum base. ***Pronotum*** (Fig. 1B) nearly circular, widest a little before middle; pronotum wider than long (PW/PL = 1.44–1.50); basal margin a little longer than anterior margin (PAW/PBW = 0.74–0.78); anterior angles slightly protruded, rounded at apex; lateral margins evenly curved, slightly constricted before posterior angles, mid-lateral setae before middle; posterior angles small, acuminate, distinctly protruded, forming evident denticle. Basal surface densely punctate, punctures reaching region between basal fovea; inner groove short and faint, a little distant from basal margin; outer groove well incised, with distinct outer margin, reaching basal margin, approximately 1/4 length of pronotum. Disc convex, smooth at middle, slightly transversely wrinkled; mid-anterior region densely punctate; lateral grooves slightly widened, distinctly punctate; median line fine but distinct. ***Elytra*** oblong, EL/EW = 1.40–1.50, widest point near middle, lateral margins subparallel before middle; basal border weakly bent forward, extending toward humeral angles; basal border and lateral margin forming an obtuse angle, with small and narrow denticle, not protruded. Basal setigerous pores absent; parascutellar striae deep as other striae, its apex joint to stria 1; all striae well incised, distinctly punctate except near to apex; punctures dense and coarse near base, gradually reducing in size to elytral apex; third interval without discal setigerous pore; ninth interval with umbilical series regularly composed of 13 pores. Elytra with isodiametric microsculpture in both sexes, quite evident on disc, very shallow near apex. Hind wings fully developed. ***Ventral side*.** Proepisternum, mesepisternum, and metepisternum heavily punctate, metepisternum lateral side twice length as basal width; abdominal sternites III–IV punctate and wrinkled except in middle, coarse area gradually narrowed from proximal sternites to distal ones; sternite VII with two pairs of marginal setae in females, with one pair in males. ***Legs*.** Male mesotibiae projection composed of two denticles on inner margin (Fig. 1C): proximal denticle larger, near middle of tibiae, with acute apex; distal one much smaller, on the middle point of the proximal denticle and distal end of tibiae. All tarsomeres with setae underside. ***Male genitalia*.** (Fig. 1D). Median lobe of aedeagus bent greater than 90 degrees; apex in lateral view gradually attenuate, a little deflected and then bent downward; dorsal margin gradually narrowed toward apex; ventral margin near straight at middle; in dorsal view, apical lamella (Fig. 1F) with maximum width subequal to length, apex rounded, slightly declined to right; apical orifice small, nearly middle placed; right paramere (Fig. 1E) long, apical half slender and gradually curved, a little thickened to apex, apex rounded without hook. ***Female genitalia*.** (Fig. 1G) Gonocoxite 2 of ovipositor very short, length a little greater than greatest width, inner and outer margin each with one ensiform seta before widest point, apex widely rounded.

#### Distribution.

Only known from the type locality in western Beijing, China. Considering it has fully-developed hind wings, this species may also be found in nearby provinces.

#### Etymology.

The scientific name of the new species comes from its type locality, Beijing.

#### Remarks.

All types of this new species were collected by the undergraduate students of Beijing Forestry University attending Forestry Cognition Field Practice in the same locality (Xiaolongmen Forest Park, Mentougou, Beijing) in late August repeatedly during the past nine years. Many of these specimens were collected by pitfall traps under (or beside) different types of forest and others were randomly hand-collected along trails. For each year, more than 100 students attend this field practical. Thus, very few of these specimens have the collector’s name recorded. Moreover, due to the difficulty to identify *Curtonotus* species in the field and mixture of specimen from various habitats, the specific habitat of the new species remains unknown for now. It is inferred that A. (C.) beijingensis sp. nov. inhabits in forest edges, like many other *Curtonotus* species found in the same area such as A. (C.) harpaloides and A. (C.) gansuensis. Although the new species is most similar to A. (C.) macronota, we hypothesize that these two species prefer different habitats and altitudes in the area around Beijing. Amara (C.) beijingensis sp. nov. was only collected in temperate broad-leaf forests above 1000 m elevation, while A. (C.) macronota was found in various open habitats in plain areas of Beijing. Besides, A. (C.) beijingensis sp. nov. possibly shares the same habitat with A. (C.) harpaloides, as both species were collected by light trap around Xiaolongmen Forest Park.

##### ﻿Supplementary notes on recorded species from eastern and central China

###### ﻿Amara (Curtonotus) gigantea species group

This species group contains only one species which is special among all Chinese *Curtonotus* for its largest body size, strongly thickened head, well-developed projection on male mesotibiae, and large apical lamella of the male genitalia.

### ﻿ Amara (Curtonotus) gigantea

Taxon classificationAnimaliaColeopteraCarabidae

(Motschulsky, 1844)

A3039D72-FC1E-5C16-AA9C-B20337D2B6C6

Fig. 2

Leirus gigantea Motschulsky, 1844: 173. (type locality: “O.-Siberia” [= east Siberia]; syntypes in ZMUM); Bates 1873: 290; Lewis 1879: 188; Andrewes 1928: 22; Matsumura 1929: 194; Kryzhanovskij 1975: 94; Sasakawa 2010: 358.
Amara herculeana Tschitschérine, 1894: 381 (type locality: “Chingan mer” [in northern China]; syntypes in ZRAS); synonymized by Kryzhanovskij 1975: 94.

#### Specimens examined.

1 male (CLYH), China, Beijing, Changping district, Hedi Road, 40.138031°N, 116.313659°E, 40 m, 2022.06.14, Yihang Li leg.; 1 male (CLYH), China, Beijing, Yuhuangmiao Village, 40.515125°N, 115.895570°E, 556 m, 2022.08.06, Yihang Li leg.; 1 male (CLHY), China, Hebei, Saihanba Forest Park, 1650 m, 2021.06, Sikai Du leg. ; 1 male (CLYH), China, Henan, Baotianman Eco-tourist Area, 2020.07, Haoyi Liu leg.; 4 males, 1 female, China, Inner Mongolia, Tongliao, Horqin Left Rear banner, Jinbaotun Town, 43.372914°N, 123.545808°E, 2022.06, Hongliang Li, leg.; 15 males, 15 females (CLHY), China, Jiangsu, Yangzhou, Jiangdu District, Heping Road, 32.377533°N, 119.574815°E, 119 m, 2023. 04, Wang leg.; 2 males, 1 female, (CBJFU), China, Jilin, Qianjin County, Jiaohe Forest Station, 43.9555°N, 127.6971°E, 397 m, 2018. 08.31–09.01, Hongliang Shi leg.; 1 male (CBJFU), China, Shaanxi, Yangxian, Huayang township, 2017.VIII.4, Weifeng Yan leg.; 1 male (CLHY), China, Zhejiang, Zhoushan, Shengsi county, Caiyuan town, 30.709870°N, 122.462928°E, 52.78 m, 2023.06.17, Haoyuan Li leg.

#### Chinese common name.

巨暗步甲.

#### Diagnosis.

**Figure 2.**
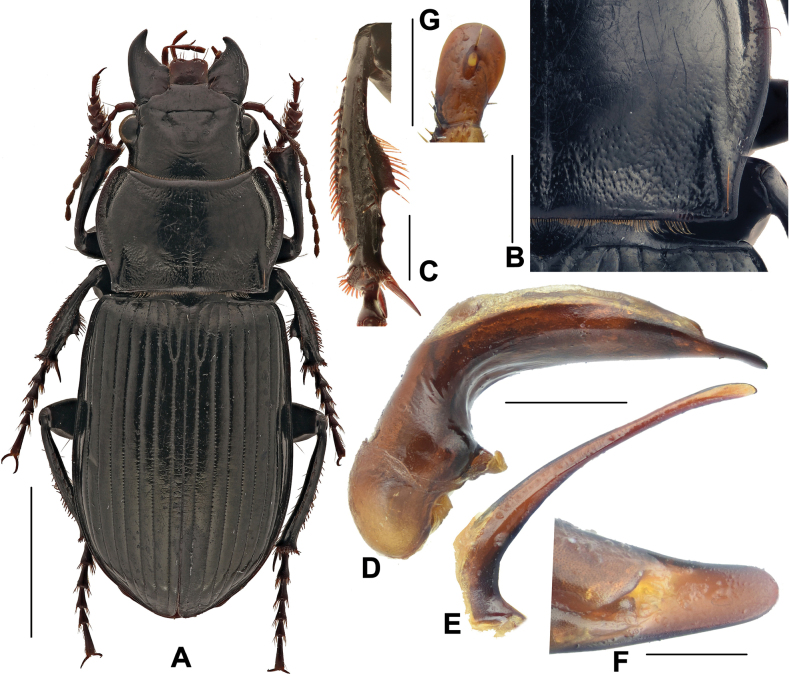
Amara (Curtonotus) gigantea**A** dorsal habitus, male (Fengning, Hebei, taken by Xiaoran Yang) **B** pronotum posterior angle (taken by Xiaoran Yang) **C** male mesotibia (taken by Xiaoran Yang) **D** lateral view of aedeagus **E** right paramere **F** apical lamella **G** female gonocoxite (Tongliao, Inner Mongolia). Scale bars: 5 mm (**A**); 1 mm (**B–E**); 0.5 mm (**F, G**).

The largest Chinese species of the subgenus, BL = 16.0–25.0 mm; dorsum black, legs dark brown to black; head strongly thickened, only a little shorter and narrower than pronotum maximum width; head with one or two supraorbital setae. Pronotum cordate (Fig. 2B), finely and densely punctate through mid-anterior, basal and lateral regions; lateral margins strongly sinuate before posterior angles; posterior angles nearly rectangular, slightly laterally protruded. Elytra elongated; lateral sides of abdominal sternites smooth. Male mesotibiae projection composed of one large denticle and two small denticles (Fig. 2C): proximal denticle widely triangular, with a row of setae, two distal denticles present between the proximal one and tibiae distal apex. Male genitalia with the apical lamella of aedeagus strongly elongate (Fig. 2F), gently declined to right, a little attenuate to tip. Gonocoxite 2 (Fig. 2G) of ovipositor elongated, length ~ 1.5× greatest width, a little widened to apex, apex widely rounded.

#### Distribution.

China (Beijing, Hebei, Gansu, Heilongjiang, Liaoning, Jilin, Inner Mongolia, Jiangsu, Sichuan, Shaanxi, Shanxi, Shanghai, Shandong, Henan*, Zhejiang, Taiwan), Japan, North Korea, South Korea, Russia (Far East, east Siberia), Oriental Region.

##### ﻿Amara (Curtonotus) tumida species group

Five species distributed in eastern China belong to this species group. They are characterized by the head with only one supraorbital seta each side and the pronotum lateral setae absent. These two distinctive features make them easily differentiated from all other *Curtonotus* species known from eastern China.

### ﻿ Amara (Curtonotus) gansuensis

Taxon classificationAnimaliaColeopteraCarabidae

Jedlička, 1957

39913625-E33A-5FF1-BC68-AC162CB844D8

Fig. 3

Amara (Curtonotus) gansuensis Jedlička, 1957: 26 (type locality: Gansu, China; holotype in NMPC); Hieke 1993: 98.
Amara (Curtonotus) pseudoseishini Hieke, 1990: 269 (type locality: “Chin Ling Shan” [= Qinling mountains, China], approx. 34°N, 108°E; holotype in ZMUC); synonymized by Hieke 1993: 98.

#### Specimens examined.

8 males, 9 females (CBJFU), China, Beijing, Mentougou District, Xiaolongmen Forest Park, 1100 m, 2014.8~2019.8; 1 female (CBJFU), China, Beijing, Huairou District, Education Center of Beijing University of Agriculture, 2016.07.30, Pingzhou Zhu leg.; 1 male, 3 females (CBJFU), China, Beijing, Songshan National Nature Reserve, 40.50806°N, 115.79111°E, 778 m, 2013.07.22–08.5, Liubo leg.; 1 female (CBJFU), China, Hebei, Chongli, CN2, 40.8814°N, 114.9499°E, 718 m, 2018. 10, Wenhao Hu leg.; 1 female (CBJFU), China, Shanxi, Gujiao, Kangjialiang Village, 37.5316°N, 112.19°E, 1230 m, 2021.09.10, Xiaojie Sun leg.; 2 females (CLYH), China, Hebei, Zhangjiakou, Hailiutu, Dayuedai, 41.176428°N, 114.512037°E, 1390 m, 2022.09.11, Cong Wang leg.

#### Chinese common name.

甘肃暗步甲.

#### Diagnosis.

**Figure 3.**
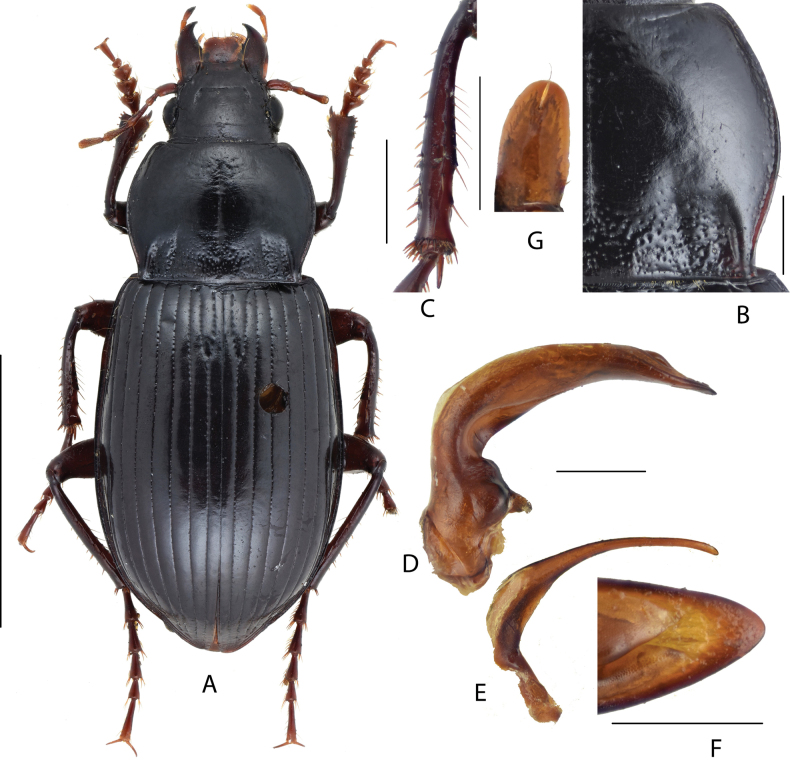
Amara (Curtonotus) gansuensis**A** dorsal habitus, male (Mentougou District, Beijing) **B** pronotum posterior angle **C** male mesotibia **D** lateral view of aedeagus **E** right paramere **F** apical lamella **G** female gonocoxite (Chongli, Hebei). Scale bars: 5 mm (**A**); 1 mm (**B–E**); 0.5 mm (**F, G**).

Medium-sized species, BL = 9.6–12.0 mm; dorsum shining black, legs reddish brown; head small, ~ 1/2 of pronotum maximum width, with one supraorbital seta. Pronotum cordate (Fig. 3B), densely punctate at base, sparsely punctate at mid-anterior region; lateral margins strongly sinuate before posterior angles; lateral grooves deeply incised, a little expanded, and distinctly punctate; posterior angles acute and laterally protruded. Elytra elongated, widest after middle; sides of abdominal sternites densely punctate. Male mesotibiae projection (Fig. 3C) composed of two small denticles; the proximal denticle acutely pointed a little beyond middle; distal denticle on the midpoint between the proximal one and tibiae apex. Male genitalia with apical lamella (Fig. 3F) elongate, subtriangular, almost straight, narrowed to apex; gonocoxite 2 (Fig. 3G) of ovipositor elongated, length ~ 1.6× greatest width, apex a little narrowed.

#### Comparison.

This species can be distinguished from the other four Chinese species belonging to this species group by its deeply incised and punctate pronotum lateral grooves.

#### Distribution.

China (Beijing, Hebei*, Gansu, Liaoning, Shaanxi, Shanxi*), North Korea, Russia (Far East).

#### Remarks.

According to our examined specimens, the pronotum of this species exhibits a variable length of the sinuation before the posterior angles.

### ﻿ Amara (Curtonotus) goniodera

Taxon classificationAnimaliaColeopteraCarabidae

Tschitschérine, 1895

4456AB4A-F0A1-5E6C-93D5-878F6BB93052

Fig. 4

Amara (Curtonotus) goniodera Tschitschérine, 1895: 164 (type locality: Korea; holotype in TMB); Hieke 1990: 256.

#### Specimens examined.

2 males (CBJFU), China, Inner Mongolia, Genhe, Daxinganling Ecological Station, 50.8061°N, 121.5824°E, 726 m, 2018.08.28, Hongliang Shi leg.; 1 male (CBJFU), China, Jilin, Baishan, Fusong County, west of Changsongling tunnel, 41.7789°N, 127.9400°E, 1577 m, 2019.08.09, Hongliang Shi & Yizhou Liu leg.; 1 male (CBJFU), China, Jilin, Antu County, north slope of Changbaishan mt, the waterfall of Tianchi, 42.0373°N, 128.0544°E, 1959 m, 2019.08.06, Hongliang Shi & Yizhou Liu leg.; 1 female (CLYH), China, Jilin, Baishan, Antu County, north slope of Changbaishan mt, the waterfall of Tianchi, 2023.06.14, Taoqi Wang leg.

#### Chinese common name.

宽瓣暗步甲.

#### Diagnosis.

**Figure 4.**
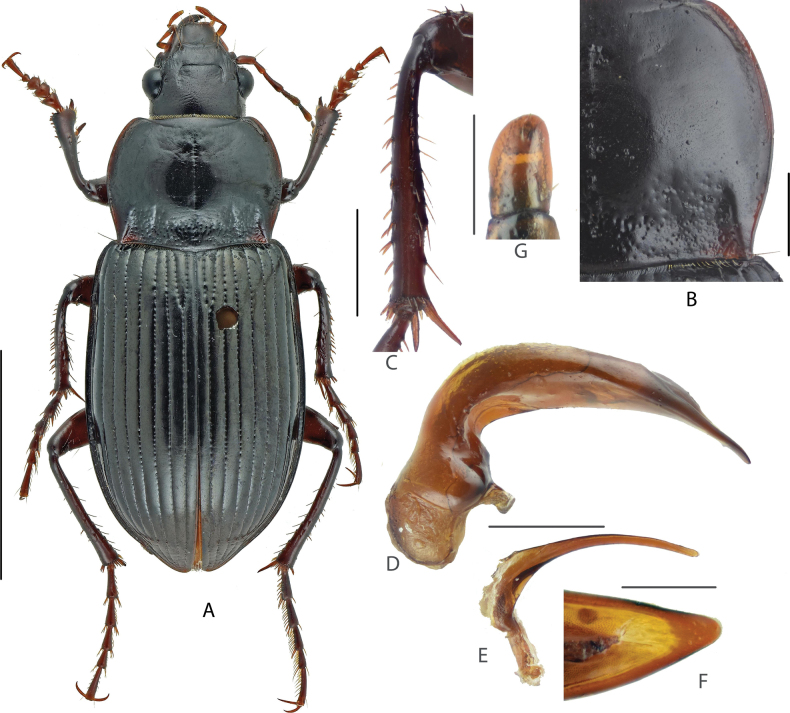
Amara (Curtonotus) goniodera**A** dorsal habitus, male (Zhangbei, Hebei) **B** pronotum posterior angle **C** male mesotibia **D** lateral view of aedeagus **E** right paramere **F** apical lamella **G** female gonocoxite (Zhangbei, Hebei). Scale bars: 5 mm (**A**); 1 mm (**B–E**); 0.5 mm (**F, G**).

Medium-sized species, BL = 10.8–12.2 mm; dorsum black, legs dark drown; head small, its width ~ 1/2 of pronotum maximum width; head with one supraorbital seta each side. Pronotum (Fig. 4B) cordate, densely punctate at base, sparsely punctate at mid-anterior region; lateral margins shallowly sinuate before posterior angles; posterior angles acute or nearly rectangular, slightly laterally protruded. Elytra elongated, widest near middle; sides of abdominal sternites densely punctate. Male mesotibiae projection (Fig. 4C) not so distinct as A. (C.) gansuensis; the proximal denticle blunt, present near apical third of tibia, distal denticles blunt and smaller than the proximal one, present at the midpoint between the proximal one and tibial distal apex. Male genitalia with apical lamella (Fig. 4F) long and straight, subpointed and narrowed at tip; gonocoxite 2 (Fig. 4G) of ovipositor elongate, broadly expanded, ~ 1.4–1.6× as long as wide, slightly attenuate to apex, apex narrowly rounded.

#### Comparison.

This species can be distinguished from the other four Chinese species belonging to this species group by having a much longer and somewhat pointed apical lamella of the median lobe. Compared with A. (C.) tumida and A. (C.) shinanensis, A. (C.) goniodera has wider and stouter gonocoxite; compared with A. (C.) gansuensis, A. (C.) goniodera, it has narrower pronotum with narrower lateral groove and shorter lateral marginal sinuation before posterior angles; compared with A. (C.) hyperborea, A. (C.) goniodera, A. (C.) goniodera has distinctly punctate abdominal sternites.

#### Distribution.

China (Gansu, Heilongjiang, Qinghai, Jilin, Shaanxi, Inner Mongolia*, Hebei*), North Korea, South Korea, Mongolia, Russia (Far East, east Siberia).

### ﻿ Amara (Curtonotus) hyperborea

Taxon classificationAnimaliaColeopteraCarabidae

Dejean, 1831

65D41756-CD61-53C4-8350-4D9630DFF938

Amara hyperborea Dejean, 1831: 800 (type locality: “Labrador” [= Newfoundland and Labrador, Canada]; holotype in MNHN); Putzeys 1865: 338; Putzeys 1866: 257; Jakobson 1906: 262; Lindroth 1953: 18; Lindroth 1954: 134, 135; Lindroth 1955: 98; Hieke 1990: 252; Hieke 1993: 147.
Curtonotus elongatus LeConte, 1850: 207 (type locality: “Lake Superior” [Probably in USA]; holotype in CUMZ); synonymized by Lindroth 1954: 134.
Leirus ovipennis Motschulsky, 1859: 156 (type locality: “Californie” [actually in western Alaska]; syntypes in ZMUM); synonymized by Hieke 1993: 147.
Leirus longicollis Motschulsky, 1860: 95 (type locality: “Daourie orientale” [in Kamchatka, Russia]; syntypes in ZMUM); Tschitschérine 1894: 389; synonymized by Putzeys 1865: 338.
Amara (Leirus) peregrina Morawitz, 1862: 258 (type locality: “Kulussutai” [in southeastern Siberia]; syntypes in ZRAS); synonymized by Lindroth 1953: 18.
Curtonotus canadensis Putzeys, 1866: 256 (type locality: “Canada boréal.” [= northern Canada]; holotype in MNHN); synonymized by Lindroth 1968: 678.
Curtonotus dejeani Putzeys, 1866: 258 (type locality: “Kamchatka”; holotype in MNHN); synonymized to A.longicollis by Tschitschérine 1894: 389.
Curtonotus pedestris Putzeys, 1866: 254 (type locality: “Udskoe Okhotsk” [= Udskoye, Region Chabarowsk, Russia; holotype in MNHN); synonymized to A. (C.) hyperborea Dejean by Hieke 1990: 252.
Curtonotus tristis Putzeys, 1866: 255 (type locality: “Oowho-Bay” [= Hudson Bay, Canada]; holotype in MNHN); synonymized to A. (C.) hyperborea by Lindroth 1968: 253.
Harpalus simulans Sahlberg, 1880: 44 (type locality: “Tschornaja ostrow” [= Yenisey range, Russia]; syntypes in NHRS); synonymized to A. (C.) hyperborea by Jakobson 1906: 262.
Curtonotus imperfectus Brown, 1930: 232 (type locality: “Bradore Bay” [in Quebec, Canada]; holotype in CMNC); synonymized to A. (C.) hyperborea by Lindroth 1954: 135.
Amara (Curtonotus) coreana Baliani, 1937: 182 (type locality: “Ompo” [= Onbo, North Hamgyeong Province, North Korea]; holotype in MSNG); synonymized to A. (C.) hyperborea by Hieke 1990: 255.

#### Chinese common name.

极北暗步甲.

#### Diagnosis.

Small- to medium-sized species, BL = 9.0–13.0 mm; dorsum brown, legs reddish brown; head small, ~ 1/2 of the pronotum maximum width; head with one supraorbital seta. Pronotum cordate, punctate at base, sporadically punctate at mid-anterior region; lateral margins nearly straight before posterior angles; posterior angles nearly rectangular, slightly laterally protruded. Elytra long, widest after middle; lateral sides of abdominal sternites smooth. Male mesotibiae projection composed of two denticles; proximal denticle acutely pointed beyond middle; distal denticle smaller, present between the proximal one and tibiae apex. Male genitalia with short apical lamella, slightly declined toward right, narrowed to apex; gonocoxite 2 of ovipositor elongated, length ~ 1.7–2.0× as greatest width, narrowed to apex.

#### Comparison.

this species can be distinguished by having relatively elongated body, impunctate sides of abdominal sternites, nearly smooth pronotum anterior portion, and nearly straight pronotum lateral edge before posterior angle.

#### Distribution.

China (Heilongjiang, Jilin, Xinjiang), Russia (Far East, east Siberia, west Siberia), Mongolia, North Korea, Europe, North America.

#### Remarks.

This species, as well as the following two species (A. (C.) tumida, A. (C.) shinanensis), was recorded from the provinces of northeastern China (Habu 1953; Hieke 1990). However, we did not find any specimens collected from eastern China that accord with these three species although several specimens of *Curtonotus* were examined from northeastern China. Considering many species of this subgenus are superficially similar externally, we suspect that these three species records from China could be based on misidentifications of similar species. We hope future research can corroborate their precise distribution in China.

### ﻿ Amara (Curtonotus) tumida

Taxon classificationAnimaliaColeopteraCarabidae

Morawitz, 1862

6E851CB0-951C-57EA-B15F-62564081F5D8

Amara (Leirus) tumida Morawitz, 1862: 258 (type locality: “Zagan-olui” [in Zabaykalsky Krai, Russia]; lectotype in ZRAS); Tschitschérine 1894: 390; Hieke 1990: 259 (lectotype designation).
Leirus tibialis Motschulsky, 1844: 343 (type locality: “Kamchatka”; holotype in ZMUM); junior secondary homonym of Amara (Amara) tibialis Paykull 1798; synonymized by Tschitschérine 1894: 390.
Amara (Curtonotus) tumida tunkunensis Hieke, 1990: 265 (type locality: “Quellgebiet des fl. Irkut” [= source of Irkut River, Buryatia, Russia]; holotype in MNHU).

#### Chinese common name.

膨胸暗步甲.

#### Diagnosis.

Small- to medium-sized species, BL = 9.0–11.0 mm; dorsum black, legs dark brown; head small, ~ 1/2 of pronotum maximum width, with one supraorbital seta. Pronotum cordate, widest near middle, densely punctate at base, sparsely punctate at mid-anterior region; lateral margins sinuate before posterior angles; posterior angles laterally protruded, acute or nearly rectangular. Elytra relatively long, widest near middle; lateral sides of abdominal sternites densely punctate. Male mesotibiae projection composed of two denticles; proximal denticle acutely pointed a little beyond middle; distal denticles smaller, present between the proximal one and tibial distal apex. Male genitalia with apical lamella slightly declined rightward, narrowed to apex; gonocoxite 2 of ovipositor elongated, length ~ 2.3× as greatest width, apex narrow.

#### Comparison.

This species is most similar to A. (C.) shinanensis, which can only be distinguished from it by having longer apical lamella, more constricted pronotum base, and relatively longer body. Compared with A. (C.) gansuensis, A. (C.) tumida has more distinct mesotibiae denticles and narrower pronotum lateral groove; compared with A. (C.) goniodera, A. (C.) tumida has more sinuate lateral margins before posterior angles, longer gonocoxite and shorter apical lamella; compared with A. (C.) hyperborea, A. (C.) tumida has punctate abdominal sternites and more constricted pronotum base.

#### Distribution.

China (Heilongjiang, Inner Mongolia), Russia (east Siberia).

#### Remarks.

According to Hieke (1990), this species has shortened wings, to ~ 1/3 the length of elytra. We could not confirm it based on current material. If this is true, it will be an important feature to distinguish A. (C.) tumida from similar species, like A. (C.) shinanensis.

### ﻿ Amara (Curtonotus) shinanensis

Taxon classificationAnimaliaColeopteraCarabidae

Habu, 1953

423F3A56-238C-5D12-9D23-7F037EB76783

Curtonotus shinanensis Habu, 1953: 43 (type locality: “Flow of the Tenryu at Iijima-mura” [in Nagano, Japan]; holotype in ITLJ); Hieke 1993: 99.
Amara (Curtonotus) seishini Jedlička, 1957: 25 (type locality: “Seishin, Olto” [= Chongjin, North Korea]; holotype in NMPC); synonymized to A. (C.) shinanensis by Hieke 1993: 99.

#### Chinese common name.

悠游暗步甲.

#### Diagnosis.

Small- to medium-sized species, BL = 9.5–11.0 mm; dorsum dark brown or nearly black, legs brown; head small, ~ 1/2 of pronotum maximum width; head with one supraorbital seta. Pronotum cordate, widest near middle, densely punctate at basal and mid-anterior regions; lateral margins sinuate before posterior angles; posterior angles slightly acute or nearly rectangular, laterally protruded. Elytra relatively short, widest near middle; lateral sides of abdominal sternites finely punctate. Male mesotibiae projection composed of two denticles (Habu 1953), which are smaller in some individuals (Hieke 1990); the proximal denticle acutely pointed a little beyond middle, distal denticles smaller, present between the proximal one and tibiae apex. Male genitalia with short apical lamella, slightly declined rightward, narrowed at apex; gonocoxite 2 of ovipositor elongated, length ~ 2× as greatest width, apex narrowed.

#### Comparison.

This species is most similar to A. (C.) tumida, which can be distinguished by having shorter apical lamella, relatively short and oval body, and wider pronotum base. To distinguish from the remaining three species, referring to comparison part of A. (C.) tumida.

#### Distribution.

China (Heilongjiang, Liaoning, Jilin), Japan, North Korea, Mongolia, Russia (Siberia).

##### ﻿Amara (Curtonotus) brevicollis species group

This group contains two species distributed in eastern China. They are characterized by a reddish brown dorsum, sometimes with a faint coppery luster, and the pronotum densely and finely punctate at anterior and lateral portions.

### ﻿ Amara (Curtonotus) dux

Taxon classificationAnimaliaColeopteraCarabidae

Tschitschérine, 1894

6098BEC9-D127-5396-8D86-5B53F430E92A

Fig. 5

Amara (Curtonotus) dux Tschitschérine, 1894: 383 (type locality: “Chingan mer.” [= south Chingan Mountains, China]; holotype in ZRAS); Hieke 1993: 100.
Amara (Curtonotus) suensoni Hieke, 1990: 249 (type locality: “Si-wan-tse” [= Xiwanzi Town, Hebei, China]; holotype in MNHU); synonymized by Hieke 1993: 100.

#### Specimens examined.

1 male (CBJFU), China, Beijing, Songshan National Nature Reserve, 40.50806°N, 115.79111°E, 778 m, 2013.08.12, Liubo leg.; 6 males, 6 females (CBJFU), Beijing, Songshan National Nature Reserve, 40.50806°N, 115.79111°E, 778 m, 2013.08.10, Bo Liu leg.; 1 male (CBJFU), Beijing, Xiaolongmen Forest Park, 1100 m, 2014.08.17–22; 1 female (CLYH), Hebei., Zhangjiakou, Zhangbei, Caoyuantianlu West Line, 41.00010926°N, 114.62708997°E, 1523 m, 2022. 09.04, Cong Wang leg.; 1 male (CLYH), Hebei, Zhangjiakou, Hailiutu, Dayuedai, 41.176428°N, 114.512037°E, 1390 m, 2022.09.11, Cong Wang leg.; 1 male (CLYH), Henan, Gongyi, Shihuiwu Village, 34.763760°N, 113.031417°E, 2022.09.07, Zheng Zhi leg.; 2 males, 1 female (CLYH), China, Gansu, Lanzhou, Yuzhong, Lanzhou University, 35.942353°N, 104.158454°E, 2022.09.22, Hanyu Yu leg.

#### Chinese common name.

点胸暗步甲.

#### Diagnosis.

**Figure 5.**
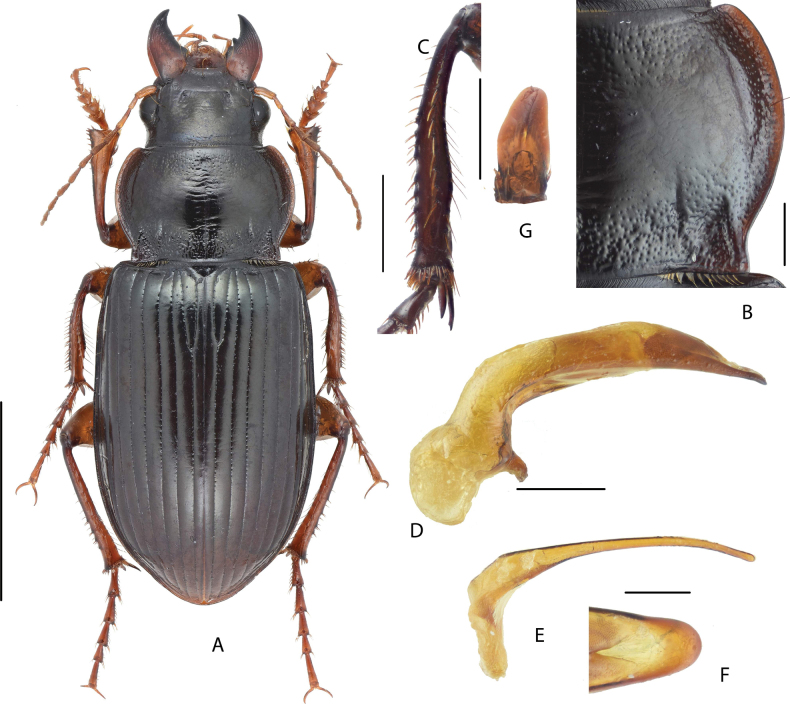
Amara (Curtonotus) dux**A** dorsal habitus, male (Gongyi, Henan) **B** pronotum posterior angle **C** male mesotibia **D** lateral view of aedeagus **E** right paramere **F** apical lamella **G** female gonocoxite (Lanzhou, Gansu). Scale bars: 5 mm (**A**); 1 mm (**B–E**); 0.5 mm (**F, G**).

Medium- to large-sized species, BL = 13.0–14.4 mm; dorsal surface dark brown, legs yellowish brown; head relatively large, slightly narrower than pronotum maximum width; head with two supraorbital setae. Pronotum (Fig. 5B) transverse, widest near middle; finely and densely punctate through basal, mid-anterior, and lateral regions; lateral margins strongly sinuate before posterior angles; posterior angles strongly laterally protruded, apex acute or near rectangular. Elytra basal border slightly curved; lateral sides of abdominal sternites sparsely wrinkled and punctate. Male mesotibiae (Fig. 5C) without distinct denticles, swollen near middle which different from females. Male genitalia with apical lamella (Fig. 5F) wide, apex rounded-truncated, indistinctly declined to right side; gonocoxite 2 (Fig. 5G) of ovipositor elongate, length ~ 2× greatest width, narrowed to apex.

#### Comparison.

This species is very similar to A. (C.) brevicollis, but can be distinguished from the latter species in having pronotum lateral margins always evidently sinuate before the acute posterior angles, pronotum mid-lateral setae present, elytra basal border slightly curved, and longer apical lamella of male genitalia. It has overlapped distribution with A. (C.) brevicollis, but is less common.

#### Remarks.

In Hieke’s work (1990), Amara (Curtonotus) suensoni, a synonym of this species, was described as having three similarly sized denticles on the male mesotibial projection. We also observed two or three very tiny denticles (much smaller than and alike the denticles in males of several other species) in our examined male specimens of this species. However, considering some female individuals also have similar-sized denticles on their mesotibiae, we do not think these additional small denticles are male-specific character.

#### Distribution.

China (Ningxia, Hebei, Beijing, Henan*, Gansu*, Liaoning, Inner Mongolia), Mongolia, North Korea, South Korea, Russia (east Siberia, Far East).

### ﻿ Amara (Curtonotus) brevicollis

Taxon classificationAnimaliaColeopteraCarabidae

Chaudoir, 1850

99BFD10E-B240-5F08-A4EE-3D5AC73E0FF2

Fig. 6

Leirus brevicollis Chaudoir, 1850: 151 (type locality: “O. Siberia” [= east Siberia]; holotype in MNHN); Lindroth 1968: 665; Hieke 1999: 170.
Curtonotus transversicollis Putzeys, 1866: 236 (type locality: “Amér. Russe.: Akina” [= Akima, Zabaykalsky Krai, Russia]; syntypes in MNHN); synonymized by Lindroth 1968: 665.
Amara (Curtonotus) kuznetzovi Lutshnik, 1928: 46 (type locality: “See Issyk-kul” [= Issyk-Kul, Kyrgyzstan]; holotype originally in Lutshnik collection, now could be lost); synonymized by Hieke 1999: 170.

#### Specimens examined.

7 males, 5 females (CBJFU), China, Beijing, Songshan National Nature Reserve, 40.50806°N, 115.79111°E, 778 m, 2013.07.21~2013.8.10, Bo Liu leg; 4 males, 4 females (CBJFU), China, Beijing, Chinese Agricultural University, 2003. 09.14, Ye Liu leg.; 2 males, 8 females (CBJFU), China, Beijing, Mentougou, Xiaolongmen Forestry Park, date between 2014.VIII-2019.VIII; 1 male (CBJFU), Beijing, Huairou, Sidaohe, 2017.VI.10–14; 1 female (CBJFU), Beijing, Shunyi, Hanshiqiao, 2016.IX.17, Zhu pingzhou leg.; 1 female (CLYH), China, Beijing, Haidian district, Shucun Park, 40.024101°N, 116.305901°E, 45 m, 2021.05, Yihang Li leg.; 1 female (CBJFU), China, Jilin, Yanbian, Helong City, 42.5506°N, 128.9951°E, 435 m, 2019.08.02, Yizhou Liu leg.; 1 female (CBJFU), China, Heilongjiang, Muleng, Ziping Mt, 2017.07.10, Zhengtong Wang leg.; 2 males, 1 female (CBJFU), China, Qinghai, Qilian County, Babao Township, 38.1804°N, 100.2454°E, 2727 m, 2019.08.16, Weifeng Yan leg., 1 male (CBJFU), China, Qinghai, Menyuan county, Xianmi, Taihua village, 37.2329°N, 102.1135°E, 2784 m, 2017.07.15, Pingzhou Zhu leg.; 2 females (CLYH), China, Gansu, Lanzhou, Yuzhong, Lanzhou University, 35.942353°N, 104.158454°E, 2022.09.22, Hanyu Yu leg.

#### Chinese common name.

短胸暗步甲.

#### Diagnosis.

**Figure 6.**
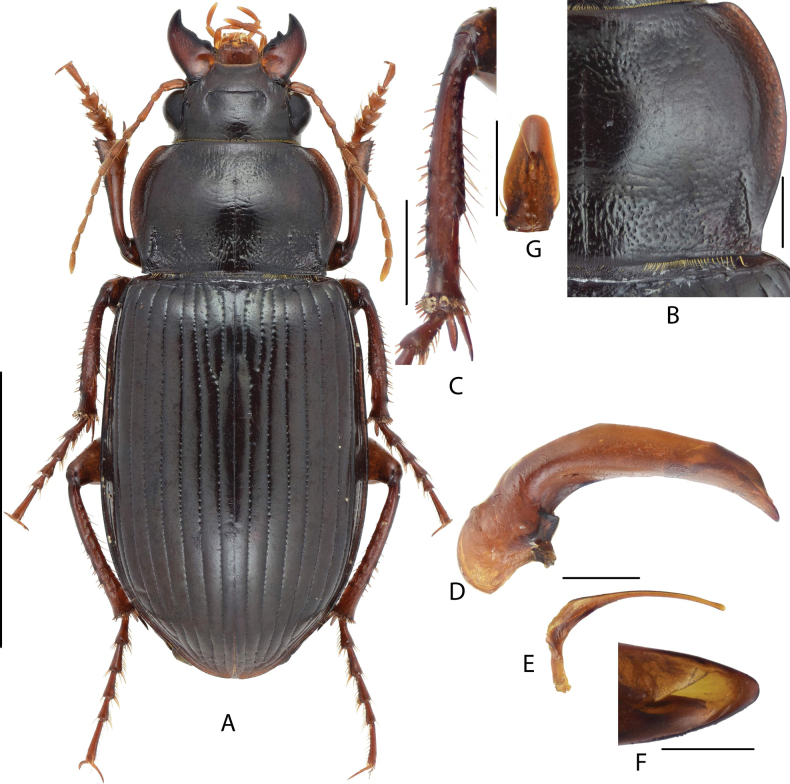
Amara (Curtonotus) brevicollis**A** dorsal habitus, male (Qilian, Qinghai) **B** pronotum posterior angle **C** male mesotibia **D** lateral view of aedeagus **E** right paramere **F** apical lamella **G** female gonocoxite (Lanzhou, Gansu). Scale bar: 5 mm (**A**); 1 mm (**B–E**); 0.5 mm (**F, G**).

Medium-sized species, BL = 9.5–12.5 mm; dorsal surface dark brown, legs yellowish brown; head relatively large, > 1/2 pronotum maximum width; head with two supraorbital setae. Pronotum (Fig. 6B) transverse, widest near middle; finely and densely punctate through basal and mid-anterior regions; lateral margins usually shallowly sinuate before posterior angles; posterior angles usually rectangular or obtuse, less protruded than the previous species. Elytra basal border nearly straight; lateral sides of abdominal sternites sparely wrinkled and punctate. Male mesotibiae projection composed of only one large denticle (Fig. 6C): proximal denticle very large, a little beyond midpoint of tibiae, significant dilated; distal denticle absent. Male genitalia with apical lamella (Fig. 6F) shorter than the previous species, a little narrowed to apex, apex rounded-triangular; gonocoxite 2 (Fig. 6G) of ovipositor elongate, length ~ 2× greatest width, apex narrowed.

#### Comparison.

This species is most similar to A. (C.) dux, but different by the absence of pronotum mid-lateral setae, presence of a distinct denticle near middle of male mesotibiae, and much shorter and narrower apical lamella of male genitalia. Besides these above, in most specimens of A. (C.) brevicollis, the pronotum posterior angles are obtuse or nearly rectangular, much less protruded than in A. (C.) dux. However, we also examined a few specimens of A. (C.) brevicollis from north China which has the pronotum outline almost identical to A. (C.) dux.

#### Distribution.

China (Beijing, Gansu, Guizhou, Hebei, Ningxia, Heilongjiang, Hubei, Jilin, Qinghai, Sichuan, Shaanxi, Xinjiang, Inner Mongolia), Russia (east Siberia, west Siberia, Far East, South European Territory), Mongolia, North Korea, South Korea, Kazakhstan, Kyrgyzstan, Turkmenistan, Europe.

##### ﻿Amara (Curtonotus) macronota species group

This species group includes six Chinese species. They are characterized by the dorsal surface being black or dark brown; head with two supraorbital setae on each side; pronotum mid-lateral setae present; pronotum impunctate or sparsely punctate on anterior portion; male mesotibiae projection varied.

### ﻿ Amara (Curtonotus) fodinae

Taxon classificationAnimaliaColeopteraCarabidae

Mannerheim, 1825

5CDE726A-6685-53A7-9455-FD7F5CFCDB15

Fig. 7

Amara fodinae Mannerheim, 1825: 20 (type locality: “Barnaul” [in Altai Krai, Ruaasia]; syntypes could be lost); Morawitz 1862: 234; Tschitschérine 1894: 387; Kryzhanovskij 1975: 92.
Leirus altaicus Motschulsky, 1844: 174 (type locality: “Altai” [in Altai Krai, Russia]; syntypes in Motschulsky’s personal collection); synonymized by Morawitz 1862: 234.
Amara (Curtonotus) primitiva Jedlička, 1957: 28 (type locality: “Quellgebiet des fl. Irkut im Ostsajan-Gebirge” [= Headwaters of the Irkut river in the east Sayan Mountains, Buryatia, Russia]; holotype in NMPC); synonymized by Kryzhanovskij 1975: 92.
Amara fodinae vicina Tschitschérine, 1894: Amarafodinaevar.vicinaTschitschérine 1894: 387 (type locality: “Amdo-Plateau: Ankhur-kashan” [in Qinghai, China]; syntypes in ZRAS).

#### Specimens examined.

6 males, 3 females (CBJFU), China, Inner Mongolia, Genhe, Daxinganling Ecological Station, 50.8061°N, 121.5824°E, 726 m, 2018.08.28, Hongliang Shi leg.; 2 males (CLYH), China, Hebei, Zhangjiakou, Zhuolu, Lingshan Scenic Spots, 40.054300°N, 115.487502°E, 1788 m, 2021.08.02, Yihang Li leg.; 1 male, 1 female (CLYH), China, Hebei, Shijiazhuang, Chang’an District, 2022.08.20–22, Ran Meng leg.; 3 males (CLYH), China, Hebei, Zhangjiakou, Zhangbei, Caoyuantian Road West Line, 41.00010926°N, 114.62708997°E, 1523 m, 2022.09.04, Cong Wang leg.; 4 males, 2 females (CBJFU), China, Hebei, Chongli, DF2, 41.0527°N, 115.3240°E, 1447 m, 2018.09, Wenhao Hu leg.; 1 female (CBJFU), China, Beijing, Mentougou District, eastern slope of Donglingshan Mt, 40.0451°N, 115.4897°E, 1710 m, 2022.07.20, Hongliang Shi & Ganyan Yang leg.; 2 male, 1 female (CBJFU), China, Beijing, Xiaolongmen Forestry park, 2014.VIII.17–22; 1 male, 2 females (CBJFU), Beijing, Songshan, 2005.8.21; 1 male (CLYH), China, Qinghai, Xining, The Party School of Qinghai Provincial committee of CPC, 36.631848°N, 101.778172°E, 2243 m, 2021.09, Bohan Cui leg.; 1 male (CBJFU), China, Qinghai, Menyuan county, Xianmi, Talihua vill. 2784 m, N37.2329 E102.1135, 2017.VII.15, Shi HL et al leg.; 1 male (CBJFU), China, Qinghai, Gangca county, Qonj Xiang, Qinghaihu bank, 3200 m, 37.1973, 99.8039, 2017.VII.19 Shi HL leg.; 1 male (CBJFU), Qinghai, Menyuan county, Meihua vill. 2784 m, N37.2574 E102.0869, 2020.VII.30 Liu YZ, Yin WQ lgt.

#### Chinese common name.

掘暗步甲.

#### Diagnosis.

**Figure 7.**
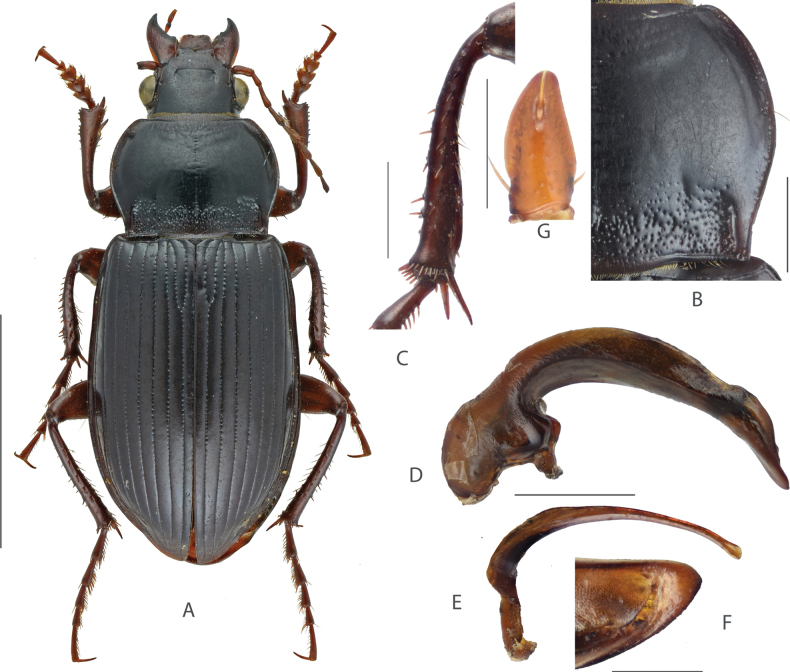
Amara (Curtonotus) fodinae**A** dorsal habitus, male (Zhangbei, Hebei) **B** pronotum posterior angle **C** male mesotibia **D** lateral view of aedeagus **E** right paramere **F** apical lamella **G** female gonocoxite (Chongli, Hebei). Scale bar: 5 mm (**A**); 1 mm (**B–E**); 0.5 mm (**F, G**).

Medium to large-sized species, BL = 11.0–13.0 mm; dorsum black or dark brown, legs usually dark brown; head relatively small, ~ 1/2 of pronotum maximum width, with two supraorbital setae. Pronotum (Fig. 7B) cordate, widest near middle; densely punctate at basal region, impunctate at mid-anterior region; lateral margins with long sinuation before posterior angles; posterior angles nearly rectangular or acute, not or slightly protruded. Elytra elongated, widest near middle, with isodiametric microsculpture; lateral sides of abdominal sternites wrinkled. Male mesotibiae projection (Fig. 7C) without defined denticle, distal half of tibiae prominently dilated. Male genitalia with short triangular apical lamella (Fig. 7F), slightly bent rightward, narrowed to apex; gonocoxite 2 (Fig. 7G) of ovipositor elongate, length ~ 2× as greatest width, distinctly attenuate to apex.

#### Comparison.

This species is different from most of Chinese *Curtonotus* by its male mesotibiae lacking denticles. From the shape of pronotum, A. (C.) fodinae is most similar to A. (C.) banghaasi, but these two species are different in many aspects: in A. (C.) fodinae the pronotum is widest near middle, but widest clearly before middle in A. (C.) banghaasi; in A. (C.) fodinae, the male mesotibiae has no distinct denticles but has two distinct denticles in A. (C.) banghaasi; in A. (C.) fodinae, the apex of apical lamella is a little acute and slightly bent rightwards, but more widely rounded and near straight in A. (C.) banghaasi; in A. (C.) fodinae, the gonocoxite 2 is strongly elongate and attenuate to apex, while in A. (C.) banghaasi it is widely rounded at apex. Some small-sized individuals of A. (C.) harpaloides also might be confused with A. (C.) fodinae, but can be differentiated by the pronotum distinctly punctate on mid-anterior region, male mesotibiae with two distinct denticles and females with gonocoxite 2 much stouter.

#### Remarks.

Two subspecies have been recognized under this species (Hieke, 1993): A. (C.) fodinae vicina is smaller and less robust and distributed in the western provinces of China, while the nominotypical subspecies is a little larger and more robust.

#### Distribution.

China (Gansu, Hebei, Beijing, Heilongjiang, Jilin, Inner Mongolia, Shaanxi, Shanxi, Qinghai, Sichuan, Xinjiang, Mongolia, Tibet), Russia (Far East, east Siberia, west Siberia), Kyrgyzstan, Kazakhstan, Tajikistan, Turkmenistan, Europe.

### ﻿ Amara (Curtonotus) banghaasi

Taxon classificationAnimaliaColeopteraCarabidae

Baliani, 1933

848A19E4-0204-55CF-AE56-723C60FF96F6

Fig. 8

Amara (Curtonotus) banghaasi Baliani, 1933: 90 (type locality: Pechino [= Peking, Beijing]; holotype in MSNG).

#### Specimens examined.

3 males, 5 females (CBJFU), China, Inner Mongolia, Hexigten Banner, Dalinuoer Lake Nature Reserve, 1200 m, 2006.07.06, Hongliang Shi leg.; 1 female (CBJFU), China, Beijing, Mentougou District, Donglingshan Mt, 40.0252°N, 115.4542°E, 1974 m, 2022. 08.22, Hongliang Shi leg.; 1 female (CLYH), China, Gansu, Zhangye, Gaotai, near Heihe, 2015.08.07–09, Deyao Zhou leg.; 1 male (CLYH), China, Gansu, Zhangye, Gaotai, Hongshahe, 2015.07.30, Deyao Zhou leg.; 1 female (CBJFU), China, Ningxia Hui Autonomous Region, Lingwu, Lingwu Farm, 2017.08.03, Yidan Zhang leg; 1 male, 1 female (CBJFU), China, Qinghai, Hainan, 36.42681°N, 100.9955°E, 3323 m, 2019.08.13, Shihao Wang leg. ; 1 male (CBJFU), China, Qinghai, Gangca, Qonj Xiang, Qinghaihu bank, 32.1973°N, 99.8039°E, 3200 m, 2017.07.19, Hongliang Shi leg.

#### Chinese common name.

棒暗步甲.

#### Diagnosis.

**Figure 8.**
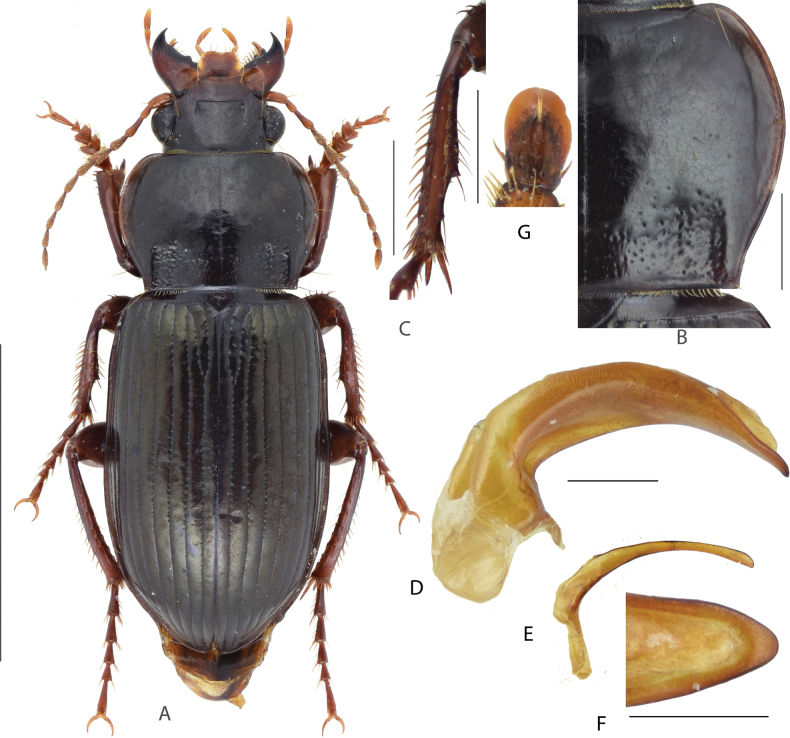
Amara (Curtonotus) banghaasi**A** dorsal habitus, male (Gangca, Qinghai) **B** pronotum posterior angle **C** male mesotibia **D** lateral view of aedeagus **E** right paramere **F** apical lamella **G** female gonocoxite (Hainan, Qinghai). Scale bar: 5 mm (**A**); 1 mm (**B–E**); 0.5 mm (**F, G**).

Medium to large-sized species, BL = 11.5–13.0 mm; dorsum black or dark brown, legs dark brown; head relatively large, more than half length of pronotum maximum width, with two supraorbital setae. Pronotum (Fig. 8B) cordate, widest near anterior third; densely and coarsely punctate at basal region, impunctate or scarcely punctate at mid-anterior region; lateral margins with sinuation long and distinct before posterior angles; posterior angles nearly rectangular or a little acute, apex not protruded. Elytra elongated, widest behind middle, with very strong isodiametric microsculpture; lateral sides of abdominal sternites sparsely wrinkled and punctate. Male mesotibiae projection composed of two denticles (Fig. 8C); proximal denticle very large, acutely pointed, a little after the middle; distal denticle much smaller, present between the proximal one and tibiae apex. Male genitalia with apical lamella shortly triangular (Fig. 8F), slightly bent leftward, narrowed to tip, apex rounded; gonocoxite 2 (Fig. 8G) of ovipositor stout, length ~ 1.5× greatest width, apex widely rounded.

#### Comparison.

This species is most similar to A. (C.) fodinae among the eastern Chinese species of Curtonotus. Comparisons between them are provided under the latter species.

#### Distribution.

China (Beijing, Heilongjiang, Hubei, Liaoning, Qinghai, Inner Mongolia*, Gansu*).

#### Remarks.

Amara (Curtonotus) daurica Motschulsky, 1844 was recorded from Heilongjiang and Qinghai provinces of China (Hieke, 2017), but there is no reliable record anywhere else in China of this species either from the literature or in our examined specimens. Due to its similar external appearance to A. (C.) banghaasi, we hesitate these records might be based on a misidentification of A. (C.) banghaasi or other similar *Curtonotus* species.

### ﻿ Amara (Curtonotus) hiogoensis

Taxon classificationAnimaliaColeopteraCarabidae

(Bates, 1873)

690C82E8-31D8-5B8A-B3C9-0EEB877C629F

Fig. 9

Curtonotus hiogoensis Bates, 1873: 291 (type locality: “Hiogo” [in Japan]; syntypes in MNHN and NHML); Lewis 1879: 190; Tschitschérine 1898: 76; Matsumura 1929: 194; Habu 1953: 41.

#### Specimens examined.

3 females (CBJFU), China, Jilin, Qianjin County, Jiaohe Forest Station, 43.9555°N, 127.6971°E, 2018.09.01, Hongliang Shi leg.; 1 male (CBJFU), China, Jilin, Antu, Erdaobaihe, 733 m, N42.4021 E128.1068, 2018.9.3, Shi Hongliang leg.; 1 female, 1 male (CBJFU), China, Hubei, Xuanen, Changtanhe, Houhe Village, 30.033006°N, 109.724061°E, 1210 m, 2017.V.7–9, Yizhou Liu leg.; 1 female (CBJFU), China, Hubei, Shennongjia, Hongping Town, 1500 m, 2013.08.15, Hao Huang leg.; 1 female (CLYH), China, Hubei, Shiyan, Zhuxi County, Shuangping Village Committee, 1151 m, 2023.06.07, Qianle Lu leg.

#### Chinese common name.

兵库暗步甲.

#### Diagnosis.

**Figure 9.**
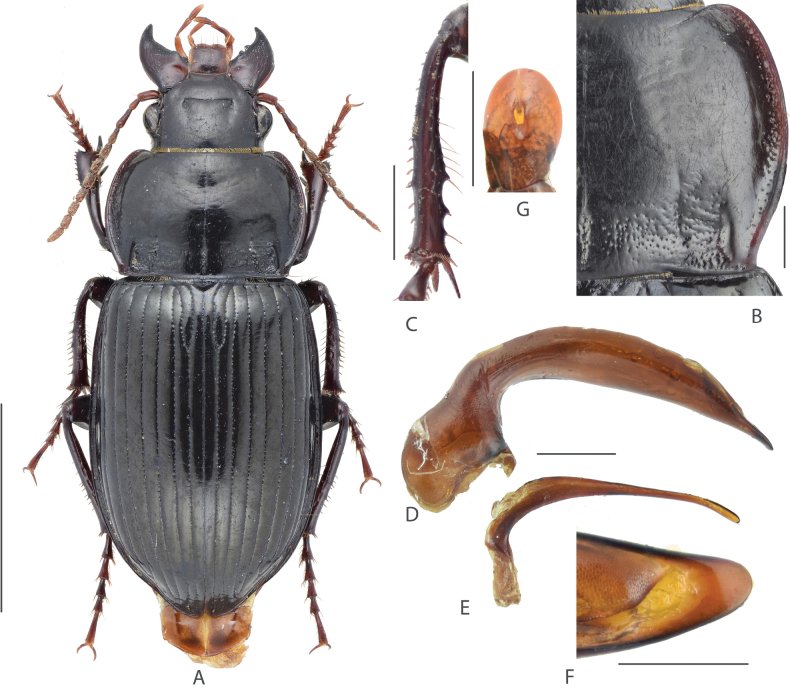
Amara (Curtonotus) hiogoensis**A** dorsal habitus, female (Jiaohe, Jilin) **B** pronotum posterior angle (Jiaohe, Jilin) **C** male mesotibia (Jiaohe, Jilin) **D** lateral view of aedeagus (Jiaohe, Jilin) **E** right paramere (Jiaohe, Jilin) **F** apical lamella (Jiaohe, Jilin) **G** female gonocoxite. Scale bars: 5 mm (**A**); 1 mm (**B–E**); 0.5 mm (**F, G**).

Large-sized species, BL = 13.5–14.0 mm; body black, legs dark brown to black; head relatively large, greater than half of pronotum maximum width, with two supraorbital setae. Pronotum (Fig. 9B) strongly transverse, slightly cordate, basal region densely punctate, mid-anterior region impunctate, sometimes finely wrinkled; lateral margins slightly sinuate near posterior angles; posterior angles wide, with indistinct denticles, barely protruded laterally; elytra oblong, widest near middle, without or only with very faint isodiametric microsculpture after middle; lateral sides of abdominal sternite punctate. Male mesotibiae projection composed of three small denticles (Fig. 9C), proximal denticle acutely pointed clearly beyond the middle, and two slightly smaller distal denticles between the proximal one and tibiae apex. Male genitalia with relatively long apical lamella (Fig. 9F), slightly bent rightward, apex widely rounded; gonocoxite 2 (Fig. 9G) of ovipositor elongate, length ~ 1.5× greatest width, apex widely rounded.

#### Distribution.

China (Anhui, Fujian, Hubei, Sichuan, Shaanxi, Zhejiang, Jilin*), Japan, North Korea, South Korea, Russia (Far East).

### ﻿ Amara (Curtonotus) harpaloides

Taxon classificationAnimaliaColeopteraCarabidae

Dejean, 1828

A22DA0CF-6355-5426-9307-85EE70A492A7

Fig. 10

Amara harpaloides Dejean, 1828: 514 (type locality: “Sibirien” [= Barnaul, Altai Krai, Russia]; syntypes in MNHN); Hieke 1999: 162.
Curtonotus convexicollis Putzeys, 1866: 232 (type locality: “Siberia”; holotype in MNHN); synonymized by Hieke 1999: 162.

#### Specimens examined.

2 males (CLYH), China, Hebei, Zhangjiakou, Zhuolu, Lingshan Scenic Spots, 40.054300°N, 115.487502°E, 1788 m, 2021.08.02, Yihang Li leg.; 1 female (CLYH), China, Beijing, Songshan National Reserve, 40.534820°N, 115.7541325°E, 1380 m, 2022.08.07, Yihang Li leg.; 1 male (CLHY), China, Beijing, Qingshui Town, Hongkou Village, light trap, 39.99464407°N, 115.48366919°E, 950 m, 2022.07.24, Haoyuan Li leg.; 19 males, 17 females (CBJFU), China, Inner Mongolia, Genhe, Greater Khingan Ecological Station, 50.8061°N, 121.5824°E, 726 m, 2018.08.28, Hongliang Shi leg.; 1 male (CLYH), China, Hebei, Zhangjiakou, Hailiutu, Dayuedai, 41.176428°N, 114.512037°E, 1390 m, 2022.09.11, Cong Wang leg.; 1 female (CBJFU), China, Gansu, Yongdeng, Liancheng Township, 36.5940°N, 102.8326°E, 1927 m, 2021. 08.04, Youyan Huang & Hanshuo Liu leg.; 1 female (CBJFU), China, Qinghai, Haixi, Tianjun County, Kuaier’ma, 37.4724°N, 98.7772°E, 3765 m, 2022.08.05, Hongliang Shi leg.; 3 males, 11 females (CBJFU), China, Qinghai, Zekong county, Maixiu forestry center, 35.2706°N, 101.9304°E, 2962 m, 2019.08.23, Weifeng Yan leg.

#### Chinese common name.

婪暗步甲.

#### Diagnosis.

**Figure 10.**
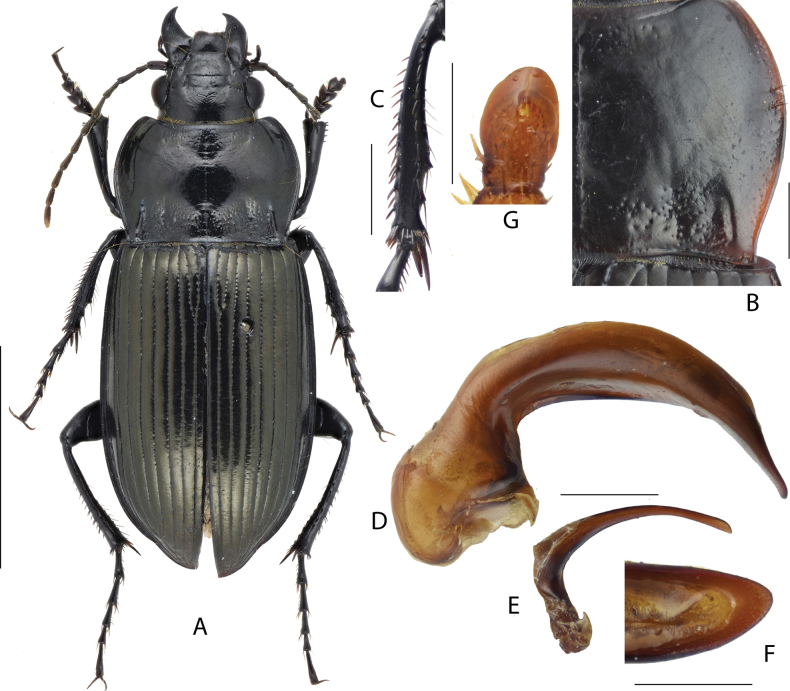
Amara (Curtonotus) harpaloides**A** dorsal habitus, male (Zhangbei, Hebei) **B** pronotum posterior angle (Tianjun, Qinghai) **C** male mesotibia **D** lateral view of aedeagus **E** right paramere **F** apical lamella **G** female gonocoxite (Yongdeng, Gansu). Scale bar: 5 mm (**A**); 1 mm (**B–E**); 0.5 mm (**F, G**).

Medium to large-sized species, BL = 10.0–12.0 mm; body black, legs dark brown to black; head small, ~ 1/2 of pronotum maximum width, with two supraorbital setae. Pronotum (Fig. 10B) subcordate, widest near middle, densely punctate at basal region, sparsely punctate at mid-anterior region; lateral margins weakly sinuate or nearly straight on posterior half; posterior angles more or less protruded laterally, nearly rectangular, apex without denticulate. Elytra oblong, widest near middle; finely punctate on basal half of striae; humeral tooth strongly and straightly protruded; elytra with isodiametric microsculpture, stronger in females; lateral sides of abdominal sternites wrinkled. Male mesotibiae projection composed of two denticles (Fig. 10C); proximal denticle acutely pointed, near apical third of tibiae; distal denticle slightly smaller and wider than proximal one, between the proximal one and tibiae apex. Male genitalia with slightly long apical lamella (Fig. 10F), straight and widely triangular, gradually narrowed to apex, apex rounded; gonocoxite 2 (Fig. 10G) of ovipositor elongate, length ~ 1.5× greatest width, apex widely rounded.

#### Comparison.

Among this species group, A. (C.) harpaloides is most similar to A. (C.) macronota and A. (C.) beijingensis, and can be distinguished from these two by having smaller elytra punctures, stronger elytral microsculpture in females, shallowly sinuate pronotum lateral margins before the posterior angles, and humeral tooth more strongly protruded.

#### Distribution.

China (Gansu, Hebei, Beijing, Heilongjiang, Qinghai, Sichuan, Shanxi, Inner Mongolia, Sichuan), Russia (west Siberia, east Siberia, Far East).

### ﻿ Amara (Curtonotus) macronota

Taxon classificationAnimaliaColeopteraCarabidae

(Solsky, 1875)

872DA695-B13A-5427-8238-A4DF39361C79

Fig. 11

Curtonotus macronotus Solsky, 1875: 265 (type locality: “Nikolskoje” [= Nikolskoye, Kamchatka Krai, Russia]; holotype in ZRAS); Matsumura 1929: 194; Lafer 1989: 180; Hieke 1995: 322; Sasakawa 2009: 107; Hieke et al. 2012: 61.
Curtonotus nitens Putzeys, 1866: 234 (type locality: “Chine boréale” [= northern China]; holotype in MNHN); Solsky 1875: 265; Lewis 1879: 189; Bates 1888: 370; Tschitschérine 1894: 385; Habu 1953: 43; Junior secondary homonym of Amaranitens Letzner, 1852.
Amara (Curtonotus) jureceki Jedlička, 1957: 29 (type locality: “Wladiwostok” [= Vladivostok, Russia]; holotype in NMPC); synonymized by Lafer 1989: 180.
Amara (Curtonotus) ovalipennis Jedlička, 1957: 30 (type locality: “Kyoto” [in Japan]; holotype in NMPC); synonymized by Hieke 1995: 322.

#### Specimens examined.

1 male (CLYH), China, Beijing, Haidian district, Baiwangshan Forest Park, 40.033893°N, 116.256957°E, 100 m, 2021.03.12, Yihang Li leg.; 2 males (CLYH), China, Beijing, Changping district, Hedi Road, 40.139454°N, 116.305624°E, 40 m, 2022.06.13, Yihang Li leg.; 3 males (CLYH), China, Sichuan, Mianning County, Tuowu Mountain, 2200 m, 2022.04.15, Yuan Li leg.; 1 female (CLHY), China, Beijing, Haidian district, Yuanmingyuan Park, 40 m, 2022.02.06, Haoyuan Li leg.; 3 males, 2 females (CBJFU), China, Beijing, Shunyi District, Hanshiqiao Wetland Reserve, 2016.10.17, Pingzhou Zhu leg.; 2 males (CBJFU), China, Shanxi, Gujiao, Yunding Mountain Preserve, 35.5246°N, 111.3553°E, 1790 m, 2021.09.10, Xiaojie Sun leg.; 1 female (CLYH), China, Hunan, Huaihua, Subaoding Mt, 1800 m, 2022. 07. 14, Yihang Li leg.; 1 male (CLYH), China, Guizhou, Changshun county, Changshun No.1 Primary School, 2020.08.25, local collector leg.; 4 males, 5 females (CCJH), China, Guangxi, Ziyuan, Shilipingtan, Zijinshan Mt, 26.169585°N, 110.499607°E, 1734 m, Jiaheng Chen leg.

#### Chinese common name.

巨胸暗步甲.

#### Diagnosis.

**Figure 11.**
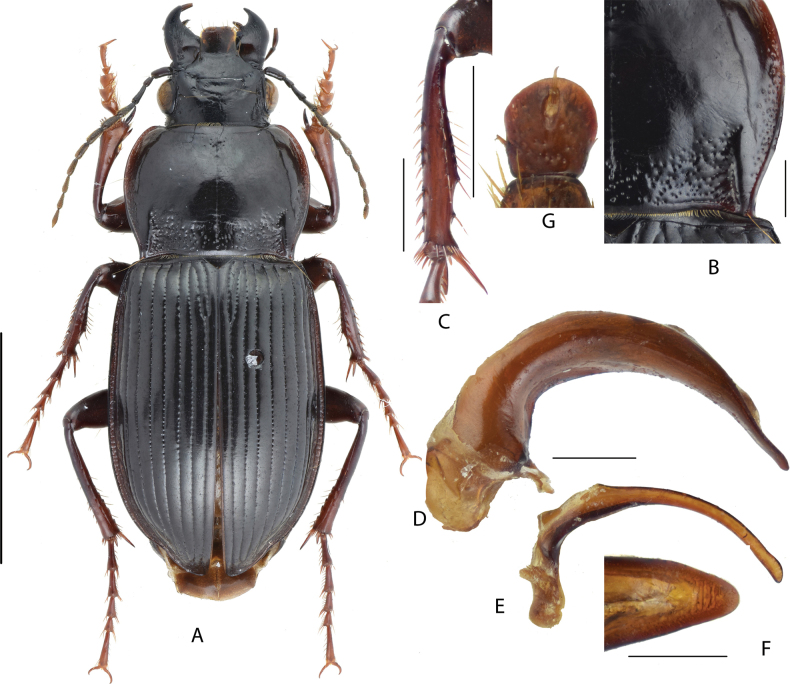
Amara (Curtonotus) macronota**A** dorsal habitus, male (Gujiao, Shanxi) **B** pronotum posterior angle **C** male mesotibia **D** lateral view of aedeagus **E** right paramere **F** apical lamella **G** female gonocoxite (Shunyi, Beijing). Scale bars: 5 mm (**A**); 1 mm (**B–E**); 0.5 mm (**F, G**).

Large-sized species, BL = 10.5–13.5 mm; body completely black, legs reddish brown to black; head medium sized, more than half length of pronotum, with two supraorbital setae. Pronotum (Fig. 11B) cordate, widest at middle; densely and very coarsely punctate at basal region, very sparsely punctate at anterior portion; lateral margins strongly sinuate before posterior angles; posterior angle strongly protruded laterally, apex sharp, nearly rectangular. Elytra oblong, widest after middle; coarsely punctate on basal two-thirds of striae; humeral tooth protruded, but smaller than the previously one, apex a little bent backward; microsculpture isodiametric in both sexes; lateral sides of abdominal sternites punctate and wrinkled. Male mesotibiae projection composed of two denticles (Fig. 11C); proximal denticle acutely pointed, near apical third of tibiae; distal denticle slightly smaller, present between the proximal one and tibiae apex. Male genitalia with relatively long apical lamella (Fig. 11F), nearly straight and widely triangular, gradually narrowed to apex, apex rounded; gonocoxite 2 (Fig. 11G) of ovipositor stout, length subequal to greatest width, apex widely rounded.

#### Comparison.

This species can be distinguished from related species by having very strongly sinuate lateral margin before posterior angles, nearly straight elytra basal border, heavy punctate on pronotum basal region and elytra striae, and very short gonocoxite.

#### Distribution.

Beijing, Fujian, Gansu, Guangdong, Guizhou, Hebei, Heilongjiang, Henan, Hubei, Jiangsu, Jilin, Jiangxi, Liaoning, Inner Mongolia, Sichuan, Shaanxi, Shanxi, Shanghai, Shandong, Tianjin, Yunnan, Zhejiang, Japan, North Korea, South Korea, Russia (Far East), Russia (east Siberia).

## ﻿Discussion

In the subgenus Curtonotus, the male modified mesotibiae projections have taxonomic importance. In some cases, closely related species can be readily distinguished by the differences on male mesotibiae. According to the examined material of macropterous *Curtonotus* species, the most basic form of the male mesotibiae is composed of an acute proximal denticle beyond the middle of tibiae, and a smaller distal denticle near the midpoint between the proximal denticle and tibial apex (e.g., Fig. 10C). This form is commonly seen in several unrelated species and thought to be plesiomorphic. In other Chinese species, the male mesotibiae are modified in different ways. It is inferred that all these different forms are derived from the basic form of a simple proximal denticle and a distal denticle.

The proximal denticle is always single but different in shape among species. In A. (C.) brevicollis (Fig. 6C), A. (C.) banghaasi (Fig. 8C), and A. (C.) macronota (Fig. 11C), a widened proximal denticle is present, with its basal margin extended to form a wide triangular projection. Among these three species, the proximal denticle is more developed in A. (C.) banghaasi than in other two. The proximal denticle in A. (C.) gigantea (Fig. 2C) is similar to the above three species, but much stronger with a sharply projected apex and serrated basal margin. In A. (C.) dux (Fig. 5C) and A. (C.) fodinae (Fig. 7C), the proximal denticle is absent, with mesotibiae slightly dilated and curved on inner margin.

The distal denticle also varies among different species. Different from the typical form with a single small acute distal denticle, A. (C.) gigantea (Fig. 2C) and A. (C.) hiogoensis (Fig. 9C) both have two equal-sized small distal denticles, whereas in A. (C.) dux (Fig. 5C), A. (C.) brevicollis (Fig. 6C), and A. (C.) fodinae (Fig. 7C), the distal denticle is completely absent.

The specialization of the middle legs is also observed in other carabid clades. *Discoderus* LeConte, a central American genus belonging to the Harpalini, has bowed mesotibiae in males (Shpeley and Ball 1978). Some species of the American harpaline genus *Stenomorphus* Dejean also have arced and hairy mesotibiae in males (Ball et al. 1991). A well-known example is *Agraschwarzeneggeri* Erwin, in which the male of this species “has an enormously enlarged middle femur” (Erwin 2002: 46).

In our study on *Curtonotus*, we put forth the hypothesis that the pattern of mesotibial denticles might be correlated with sexually antagonistic selection within a species. Through our observations of *Curtonotus* copulation images, we observed that certain species (e.g., Amara (C.) aulica Panzer) prefer to mate on stalks or flowers, posing a risk of the male falling off. During this process, the male mesotibiae constantly grip the female’s elytra lateral borders or shoulders. This adaptation becomes essential as in most carabid beetles the outer edge of elytra border forms a minute upward reflex, and the large denticles on the male’s mesotibiae facilitate securing the female’s body by grasping her elytral border reflex, preventing any mishap if the female struggles during mating. Moreover, it may also enable the male to protect the female from disturbances by other males.

Similar functions have been observed in various insect clades, such as the well-known example of the water strider genus *Rheumatobates* Bergroth (Gerridae, Hemiptera), where males possess specialized antennae to grasp and control females during mating (Khila et al. 2012). In the case of the blister beetle *Linsleyaconvexa* Leconte (Meloidae, Coleoptera), males have spinose tubercles at the base of each foreleg femur, as well as shortened foreleg tibiae and reduced foreleg tibial spurs, all regarded as adaptations for clasping the female and supporting the male’s body during mating (Selander and Pinto 1967). Similarly, within the Adephaga, the tiger beetle genus *Manticora* Fabricius exhibits large and asymmetrical mandibles in the males, which are used to guard the female by grasping her thorax (Oberprieler and Arndt 2000).

Among *Curtonotus* species, the one with the largest denticle is A. (C.) gigantea, which also has the largest size and a robust body, indicating more difficulty in controlling the female. The presence of large denticles in males may assist in better control over females. Conversely, the *Curtonotus* species in the *tumida* group, having smaller body sizes within the subgenus, display less specialized mesotibiae. This conclusion might be applicable to other *Amara* species with mesotibiae denticles, such as members of the subgenus Bradytulus. However, species like A. (C.) dux and A. (C.) fodinae have medium to large body sizes but lack denticles. We hypothesize that the independent loss of denticles in A. (C.) dux and A. (C.) fodinae may have occurred due to differing behavior strategy, reducing the selective pressure of competition between the opposite sexes. Nevertheless, the specific evolutionary dynamics behind this behavior require further exploration.

It is essential to consider that the specialized male mesotibiae might serve multiple functions during the mating process. For instance, the unique shape of male mesotibiae may serve as a recognition tool for females, preventing copulation between different species. The denticles may also provide a species-specific, localized tactual stimulus for the female, as observed in the blister beetle (Selander and Pinto 1967). Due to limited materials, we have not examined many other species, especially those with distributions outside of China. Additionally, there is scarce documentation of the mating process of *Curtonotus* species. We hope that future research can encompass a broader range of *Curtonotus* species and carefully observe their mating process to further substantiate our hypothesis.

## Supplementary Material

XML Treatment for Curtonotus

XML Treatment for Amara (Curtonotus) beijingensis

XML Treatment for Amara (Curtonotus) gigantea

XML Treatment for Amara (Curtonotus) gansuensis

XML Treatment for Amara (Curtonotus) goniodera

XML Treatment for Amara (Curtonotus) hyperborea

XML Treatment for Amara (Curtonotus) tumida

XML Treatment for Amara (Curtonotus) shinanensis

XML Treatment for Amara (Curtonotus) dux

XML Treatment for Amara (Curtonotus) brevicollis

XML Treatment for Amara (Curtonotus) fodinae

XML Treatment for Amara (Curtonotus) banghaasi

XML Treatment for Amara (Curtonotus) hiogoensis

XML Treatment for Amara (Curtonotus) harpaloides

XML Treatment for Amara (Curtonotus) macronota

## ﻿Additional information

### Author ORCIDs

Yihang Li https://orcid.org/0000-0001-5677-0440

Haoyuan Li https://orcid.org/0009-0005-0314-9542

Hongliang Shi https://orcid.org/0000-0002-9989-5830

### Data availability

All of the data that support the findings of this study are available in the main text.
